# How Do Nepotism and Favouritism Affect Organisational Climate?

**DOI:** 10.3389/fpsyg.2021.710140

**Published:** 2022-01-07

**Authors:** Jolita Vveinhardt, Rita Bendaraviciene

**Affiliations:** Department of Management, Faculty of Economics and Management, Vytautas Magnus University, Kaunas, Lithuania

**Keywords:** nepotism, favouritism, organisational climate, employee behaviour, manager behaviour

## Abstract

This study seeks to determine the effect of nepotism and favouritism on organisational climate. Using the method of random sampling, 269 persons working in Lithuanian organisations were surveyed. The received data was analysed *via* the application of the methods of correlation and linear regression. It was determined that organisational climate is influenced significantly by variables such as the manager’s behaviour, safety and relationships with employees, values and traditions, communication, sharing of information, behaviour of employees, and interrelationships and tolerance of one another. Meanwhile, nepotism and favouritism are influenced by the lower number of climate variables (fear related to the absence of concreteness and security, such as joining an organisation, union and tolerance of individuals who have shared interests). This work fills the void in the knowledge of connections that nepotism and favouritism have with organisational climate, drawing attention to the mutual interaction between these phenomena. The article presents a discussion and the research limitations, and provides guidelines for further research.

## Introduction

If nepotism and favouritism are a natural phenomenon (Salter, 2002; Rice et al., 2010; Salter and Harpending, 2013), then perhaps it is not worth it for organisations to fight it at all. On one hand, even though nepotism and favouritism are often evaluated negatively, some authors see their benefit in the context of social connections. For instance, Spranger et al. (2012) claim that some organisations can function successfully at a certain level of nepotism which does not harm the organisation’s understanding of justice, while Jones and Stout (2015) think that “social connections in some crony relationships and apparently nepotic ones may add considerable value to organisations” (p. 2). Other authors note that social connections can be beneficial in the context of employment (Horak, 2018), they have a positive impact on one’s attitude toward work (Song and Olshfski, 2008), and can bring benefit to the performance results of family business companies because of organisational social capital (Schmid and Sender, 2019). Moreover, Hildreth et al. (2016) researched the connections between ethical behaviour and the loyalty associated with nepotism. Their study showed that individuals who were more loyal to their fraternities and study groups were deceitful less frequently than their less loyal colleagues. The results of another study demonstrated that such connections help one create a psychological contract and motivate one more to maintain respect toward one’s manager, which satisfies the interests of both the manager and the subordinate (Shaheen et al., 2019).

On the other hand, numerous authors are sceptical toward the existence of nepotism and favouritism in organisations based on the evidence of their negative impact on the organisation’s activities (e.g., Haugen and Westin, 2016; Elbaz et al., 2018; Bilal et al., 2020). Admitting that nepotism and favouritism helped increase the possibilities of survival or reproduction in the process of evolution, Li et al. (2018) claim that modern contexts are very different from the environment which existed during the development of the humans’ psychological mechanisms. While Pearce (2015) does compliment Jones and Stout’s (2015) contribution to the explanation of nepotism’s role, he associates the positive conclusions with insufficient evaluation of evidence. According to the author, not all employees are capable of transparently avoiding the pressure of obligations to family, while patronage based on simple extra organisational personal relations is very dangerous. It is thought that what is considered a virtue or a risk is highly dependent on the perspective from which it is seen, e.g., different participants, different organisational and societal level (Haugen and Westin, 2016, p. 84). Furthermore, it is noted that, depending on the culture and its values, the evaluation of nepotism itself differs as well (Im and Chen, 2020).

Although there is no abundance of studies that would systematically research the connection between organisational climate and nepotism and favouritism, a look at individual variables of organisational climate reveals rather contradictory research results. For instance, while the friendliness of employees and managers itself is associated with a strong organisational climate (Herman et al., 2008), the non-beneficiaries experience a sense of insecurity (Neckebrouck et al., 2018; Arasli et al., 2019). Though paternalism can turn into nepotism, a study by Erben and Güneşer (2008) uncovered that benevolent paternalistic leadership had a “moderate effect on affective commitment but a strong effect on continuance commitment” (p. 966). Yet, another study showed that while nepotism was negatively associated with satisfaction about one’s job, it did not have significant influence on emotional obligation (Daskin et al., 2015). Thus, evidently, there is a demand for a systematic perspective, i.e., to evaluate how nepotism and favouritism are related to individual variables of organisational climate. It should also be noted that so far there is little data on how these phenomena pertain to these variables in organisations operating in Eastern and Central Europe (Sroka and Vveinhardt, 2020). Therefore, the aim of this study is to determine the impact of nepotism and favouritism on organisational climate.

## Theoretical Background

### Organisational Climate

Organisational climate is named as one of the most important aspects of the organisational environment, which is directly related to employee behaviour (Berberoglu, 2018). From the standpoint of Glisson and James (2002), these are perceptions of individuals, describing the work environment, which can be generalised. In other words, these perceptions reflect a common attitude to the organisational policy and procedures (Sethibe and Steyn, 2016). According to Ostroff et al. (2012), the original Lewinian basis for climate has been expanded to include theoretical perspectives on interactivity and cognition. In other words, climate was understood as a set of descriptions of organisational features, events, and processes based on perceptual principles (p. 651). Based on climate research, Anderson and West (1998) distinguished at least two approaches. The first, the cognitive schema approach, defines climate as individuals’ constructive representations. The second, the shared perceptions approach, expresses a common understanding of the organisation’s policies and practices, procedures. Meanwhile, the “interactive” approach includes interaction between group members as a key determinant of the organisational climate (Moran and Volkwein, 1992).

### Behaviour of Managers

As the climate affects the interaction between management and employees (Momeni, 2009; Rostila et al., 2011), depending on the prevailing leadership style, different emotional and behavioural reactions of employees can be expected (Koene et al., 2002; Işçi et al., 2015). For example, the development of a caring climate has a direct impact on job satisfaction and positive work outcomes (Fu and Deshpande, 2014), while a poor organisational climate can be linked to abusive supervision, which causes stress, psychological distress, and silence for employees (Zhang and Bednall, 2016; Park et al., 2018; Wu et al., 2018). However, the quality of the managers’ conduct does not only directly affect them. Richard et al. (2020) found that abusive supervision promotes aggression among power-oriented individuals when the human resource support climate is weak. This leads to conflicting behaviour, creating an additional negative effect that makes employees feel insecure. The sense of security provided by an atmosphere of trust and support (Anderson and West, 1998) is also associated with close monitoring (Rietzschel et al., 2014), but the reactions to it vary depending on personal characteristics. For example, close monitoring had negative effects on job satisfaction and motivation for employees who tended to have greater autonomy, whereas those with less autonomy were not affected (Rietzschel et al., 2014).

### Organisation’s Assessment

Climate expresses the employees’ attitude toward organisational circumstances and how they respond to psychological interests related to personal well-being (Jones and James, 1979; Rostila et al., 2011; Schneider et al., 2013). Employees expect fair compensation from the organisation for their contributions, and so perceptions of procedural justice and reward relate to the employees’ role performance (Chen et al., 2016). A study by Shih and Chuang (2013) showed that fair evaluations and compensation systems can be useful for an organisation to demonstrate compliance between obligations and rewards. When employees realise that the employee-caring organisational climate covers all employees, they also adapt more easily to broken promises, if the latter are unavoidable.

In addition, a meta-analysis by Zohar and Luria (2005) confirmed that the overall approach to work acts as a mechanism, partly explaining the relationship between the type of competing values framework climate and the results of work. Although values are usually studied in the context of organisational culture, Schneider et al. (2013) believes that climate researchers can evaluate not only policies, practices, and procedures, but also values. This is what they can mean to members of an organisation and how they are conveyed.

### Employee Interrelationships

Studies show that employees’ perceived safety and psychological well-being are strongly linked to an organisation’s ability to ensure healthy employee-to-employee procedures (Dollard et al., 2017; Einarsen et al., 2018; Nielsen and Einarsen, 2018). Clear management procedures allow employees to experience a sense of definiteness and security. For example, ethical leadership has been found to reduce uncertainty by creating a psychologically secure climate for members, thereby encouraging them to act creatively, whereas quality communication and information sharing creates a favourable environment for the smooth pursuit of the organisation’s goals (Anderson and West, 1998). In addition, employees feel safe when they perceive that they will not be alienated, co-workers respect their views and competence, are interested in them as individuals, and are able to resolve conflicts constructively (Edmondson, 1999). Meanwhile, interpersonal conflicts are associated with perceptions of mutual incompatibility, irritability, and frustration in relation to co-workers (Jehn and Mannix, 2001). According to them, members of teams that have a perfect conflict profile possess similar predetermined value systems, a high level of trust and respect, and norms of open discussions related to conflicts.

### Internal Policy and Norms of Behaviour Within an Organisation

On the one hand, respect for co-workers and tolerance of individual differences (health, gender, race, etc.) are associated with lower levels of stress (Matt and Butterfield, 2006), health and well-being (Harris et al., 2018), on the other hand, the ability to tolerate is associated with the employee’s own high levels of emotional stability (Beus et al., 2015). However, whether negative attitudes toward “different” persons turn into violent acts depends on the policy of the organisation that tolerates discrimination (Vogt et al., 2007; Harris et al., 2018). Studies show that an organisation’s antidiscrimination climate, which includes receiving complaints, dealing with complaints and sanctions, can protect against negative actions (Tenbrunsel et al., 2019). In addition, according to Dickson et al. (2006) a general approach toward the policies, practices and norms of an organisation that refer to mechanistic or organic organisational forms makes it possible to evaluate the organisation’s approach and perception of the organisation’s form.

### Nepotism, Favouritism, and Climate

Although traditionally nepotism is perceived as a demonstration of favouritism toward family members during the recruitment process or during promotion (Pelletier and Bligh, 2008, p. 828), some authors associate this phenomenon with discrimination (e.g., Jones et al., 2008; Erden and Otken, 2019; Hoang and Huynh, 2020). That is, with the restriction and inequality of opportunities for some employees, which is based on certain social norms, when one group is shown favour and patronage, the rest perceive it as unjust behaviour. Colquitt et al. (2001), summarising many studies, notes, what is called right is based on a subjective understanding of fairness and distinguishes two types of justice.

The first type is defined as fairness of outcome distributions or allocations, whereas the second one is described as fairness of the procedures used for outcome distributions (p. 425). If employees perceive that the procedures and policies used by the organisation are not applied to everyone in a uniform and consistent manner, a negative attitude toward the integrity of the organisation develops (Mohammad et al., 2019). Studies show that perceived dishonesty and injustice of an organisation are associated with both high levels of nepotism and favouritism (Dickson et al., 2012; Sonnentag, 2012; Jones and Stout, 2015) and a poor organisational climate (Shin, 2012; Chernyak-Hai and Tziner, 2014). In addition, a study by Daskin et al. (2015) revealed that nepotism as an organisation climate variable was associated with intrinsic motivation. External motivation is defined as the performance of an action due to the utility of its perception toward instrumental and functional value, whereas internal motivation is defined as the performance of an action for pleasure associated with satisfying different psychological needs (Li and Wen, 2019, p. 3).

## Materials and Methods

### Sample

The concept of this quantitative research was based on studies of organisational climate, nepotism, and favouritism that were conducted previously in different countries. However, to the authors’ knowledge, until now, the connection between organisational climate, nepotism, and favouritism has only been researched according to separate variables, which highlighted the need for a systematic perspective. Research shows that nepotism and favouritism is a rather frequent phenomenon in postcommunist countries of Central and Eastern Europe (e.g., Onoshchenko and Williams, 2014; Ignatowski et al., 2019); for this reason, research was conducted by surveying persons who work in organisations of Lithuania.

### Procedures

Research was conducted using the method of random sampling by presenting the respondents with an online questionnaire form. The questionnaire was restricted from repeated filling-in; it was also not possible to submit an incomplete questionnaire, which served as protection against skipped questions. The respondents received explanations of the goal and ethics of the research, and they were guaranteed anonymity and confidentiality. As this study is exploratory, its main purpose was to test the questionnaire in a relatively small sample. Therefore, the sample size is based on Comrey and Lee’s (2016) proposed graded scale of sample sizes for scale development, according to which the sample is considered fair when it includes 200 respondents, and the sample is considered good when it includes 300 respondents. In this case, our sample falls between the categories of fair and good; i.e., the survey included 269 respondents.

### Measures

The survey was conducted in Lithuania using the questionnaire “Nepotism and Favouritism in the Context of Revealing the Organisational Microclimate” (NFOM). The questionnaire’s initial version NFOM-125 encompassed 125 statements in total, 44 of which were dedicated to diagnosing nepotism and favouritism, while the remaining ones were for diagnosing the climate. The questionnaire’s initial version NFOM-125 consisted of 4 scales and 15 subscales. This version of the questionnaire, which is shortened, includes 4 scales, 12 subscales, and 114 statements, 39 of which are dedicated to diagnosing nepotism and favouritism. The questionnaire’s psychometric characteristics have been tested both in its full version (NFOM-114 items), and after separating the statements on nepotism and favouritism from the statements on climate (N&F-39). Significantly, the questionnaire has been found to have high psychometric reliability characteristics. For instance, in the questionnaire “Nepotism and Favouritism in the Context of Revealing the Organisational Microclimate,” the Cronbach’s alpha values ranged between 0.77 and 0.94, whereas Spearman-Brown values, which are typically lower, ranged between 0.68 and 0.92. This demonstrates high internal compatibility and stability of the scales. Slightly lower, but nevertheless high internal compatibility and stability of the scales was also demonstrated by the test of the questionnaire “Nepotism and Favouritism in the Organisations.”

## Results

The study involved working respondents aged from 18 to retirement age, the majority of whom were persons under 40 (83.6%). Two thirds of respondents have worked in their current workplace for 1–7 years, the majority (74%) have had higher education. Men constituted 26%, and women, 74% of the total study sample. Almost two thirds of the respondents (65.4%) were employed persons aged under 30, many of whom were born after the reestablishment of the country’s independence. Four-fifths of the respondents had already acquired higher education degrees, whereas about a half had worked for longer than 3 years, i.e., had sufficient work experience. Notably, most of the respondents worked in local capital companies, which demonstrates national tendencies in the capital structure and management traditions.

Considering the interaction between climate, nepotism, and favouritism at the scale level, correlation links that differ in strength but in all cases are statistically significant can be seen. In this case, two scales of nepotism and favouritism distinguish themselves. For example, strong relationships (0.6 < *r* < = 0.8) were found between factors related to behaviour of managers, monitoring and security, and organisational microclimate (FRBM *r* = 0.674, *p* < 0.01; FROA *r* = 0.679, *p* < 0.01; FREI *r* = 0.685, *p* < 0.01) as well as between factors related to employee interrelationships and all factors of organisational microclimate, where the highest value is (FREI; *r* = 0.771, *p* < 0.01, whereas the relatively lowest, FRIP *r* = 0.654, *p* < 0.01). Meanwhile, moderate correlations exist between factors related to the organisation’s assessment, factors related to internal policy and norms of behaviour within organisation and all factors of organisational microclimate (Table 1). A more detailed view is revealed at the subscale level (Table 2).

**TABLE 1:** Correlative links between climate, nepotism, and favouritism (at scale level) (N_min_ = 269; N_max_ = 269).

Scales	Organisational Microclimate			
Nepotism and Favouritism	FRBM	FROA	FREI	FRIP
FRBM-N&F. Factors related to behaviour of managers, monitoring and security	0.674**	0.679**	0.685**	0.546**
FROA-N&F. Factors related to the organisation’s assessment	0.582**	0.641**	0.642**	0.554**
FREI-N&F. Factors related to employee interrelationships	0.714**	0.673**	0.771**	0.654**
FRIP-N&F. Factors related to internal policy and norms of behaviour within organisation	0.515**	0.511**	0.554**	0.559**

**Correlation is significant at the 0.01 level (2-tailed).

*Spearman correlation coefficient.*

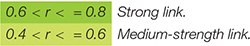

**TABLE 2:** Correlative links between climate, nepotism, and favouritism (at subscale level) (N_min_ = 269; N_max_ = 269).

Subscales	Organisational Microclimate										
	
	CS. Fears related to the lack of certainty and security	AE. Achievements and evaluations	VT. Values and traditions: fostering of ideology	ED. Organisational entry, downgrading and dismissal	CI. Communication and information sharing	MB. The managers behaviour and relationships with employees	SM. Supervision, monitoring and checking of activity and responsibility	EB. Employee behaviour and interrelationships	UP. Unification of persons sharing common interests, attitudes and objectives	CC. Confrontation of conflicting interests, attitudes and objectives	TD. Tolerating “different” persons
**Nepotism and favouritism**	**CS**	**AE**	**VT**	**ED**	**CI**	**MB**	**SM**	**EB**	**UP**	**CC**	**TD**

MB-N&F. The manager’s behaviour and relationships with employees	0.672**	0.617**	0.586**	0.629**	0.649**	0.657**	0.472**	0.614**	0.625**	0.644**	0.546**
AE-N&F. Achievements and evaluations	0.499**	0.555**	0.518**	0.541**	0.573**	0.519**	0.430**	0.524**	0.532**	0.501**	0.518**
VT-N&F. Values and traditions: fostering of ideology	0.430**	0.514**	0.443**	0.502**	0.548**	0.467**	0.406**	0.499**	0.505**	0.512**	0.476**
CI-N&F. Communication and information sharing	0.507**	0.487**	0.479**	0.530**	0.673**	0.559**	0.546**	0.633**	0.583**	0.562**	0.558**
EB-N&F. Employee behaviour and interrelationships	0.564**	0.604**	0.579**	0.611**	0.658**	0.675**	0.571**	0.666**	0.656**	0.711**	0.643**
TD-N&F. Tolerating “different” persons	0.409**	0.429**	0.477**	0.448**	0.484**	0.526**	0.382**	0.461**	0.470**	0.455**	0.505**
OP-N&F. View from the organisation’s perspective	0.265**	0.292**	0.188**	0.283**	0.261**	0.258**	0.260**	0.254**	0.366**	0.318**	0.325**

**Correlation is significant at the 0.01 level (2-tailed).

*Spearman correlation coefficient.*

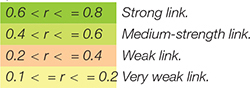

Importantly, only the view from the organisation’s perspective has weak links to almost all subscales of the organisation’s climate, while the supervisors’ and employees’ behaviour as well as their interrelationships stand out the most. In this case, strong correlative links have been identified between the subscale of manager’s behaviour and relationships with employees, which represents nepotism and favouritism, and such climate subscales as communication and information sharing (*r* = 0.649, *p* < 0.01), the manager’s behaviour and relationships with employees (*r* = 0.657, *p* < 0.01), and fears related to the lack of certainty and security (*r* = 0.672, *p* < 0.01). Moreover, it was found that employee behaviour and interrelationships (nepotism and favouritism) have strong correlative links to communicative environment (*r* = 0.658, *p* < 0.01), management behaviour (*r* = 0.675, *p* < 0.01), the quality of the employees’ own interrelationships (*r* = 0.666, *p* < 0.01), and incompatibility of interests, views, and goals (*r* = 0.711, *p* < 0.01) (Table 2). Nevertheless, the ways in which nepotism, favouritism, and climate affect one another are demonstrated by the results of regression analysis (Table 3). The results of the validation show not only that the variables representing nepotism and favouritism (except for achievements and evaluations and view from the organisation’s perspective, whose statistical reliability has not been confirmed) interact with the climate (*r* = 0.830), but also that the climate influences nepotism and favouritism (*r* = 0.831). The value of the coefficient of determination *r*^2^ = 0.689 shows that 68.9% of changes in the dependent variable organisational microclimate are determined by changes in the independent factors of nepotism and favouritism. Organisational microclimate affects nepotism and favouritism also very similarly (*r*^2^ = 0.691). Thus, it can be concluded that all independent variables explain as much as 69.1% of the part of dispersion of the dependent variable. It should be noted that in the latter case, the impact of such factors as the manager’s behaviour and relationships with employees, supervision, monitoring, and checking of activity and responsibility, achievements and evaluations, values and traditions, communication and information sharing, employee behaviour and interrelationships, confrontation of conflicting interests, attitudes and objectives was not statistically significant (Table 3).

**TABLE 3:** Links between organisational climate, nepotism, and favouritism.

	Dependent variable – Organisational Microclimate (OM)	*R*	*R* ^2^	*R*^2^ *revised*
		**0.830**	**0.689**	**0.681**

	**Independent variable – Nepotism and Favouritism (N&F)**	**Non-standardised Beta coefficient**	**Standardised Beta coefficient**	***t***

	(Constant)	0.512		3.297***
MB-N&F. The manager’s behaviour and relationships with employees	The manager’s behaviour and relationships with employees	0.180	0.229	4.330***
AE-N&F. Achievements and evaluations	Achievements and evaluations	0.042	0.037	0.746
VT-N&F. Values and traditions: fostering of ideology	Values and traditions: fostering of ideology	0.103	0.099	1.948
CI-N&F. Communication and information sharing	Communication and information sharing	0.153	0.195	3.915***
EB-N&F. Employee behaviour and interrelationships	Employee behaviour and interrelationships	0.285	0.333	6.064***
TD-N&F. Tolerating “different” persons	Tolerating “different” persons	0.145	0.155	3.736***
OP-N&F. View from the organisation’s perspective	View from the organisation’s perspective	−0.042	−0.042	−0.954

	**Dependent variable – Nepotism and Favouritism (N&F)**	***R***	***R* ^2^**	***R*^2^ *revised***

		**0.831**	**0.691**	0.678***

	**Independent variable – Organisational Microclimate (OM)**	**Non-standardised Beta coefficient**	**Standardised Beta coefficient**	***t***

	(Constant)	0.705		6.134***
CS. Fears related to the lack of certainty and security	Fears related to the lack of certainty and security	0.090	0.128	2.122
MB. The manager’s behaviour and relationships with employees	The manager’s behaviour and relationships with employees	0.065	0.092	1.272
SM. Supervision, monitoring and checking of activity and responsibility	Supervision, monitoring and checking of activity and responsibility	0.007	0.010	0.186
AE. Achievements and evaluations	Achievements and evaluations	0.026	0.036	0.574
VT. Values and traditions: fostering of ideology	Values and traditions: fostering of ideology	−0.012	−0.016	−0.249
ED. Organisational entry, downgrading and dismissal	Organisational entry, downgrading and dismissal	0.194	0.237	4.289***
CI. Communication and information sharing	Communication and information sharing	0.112	0.149	1.788
EB. Employee behaviour and interrelationships	Employee behaviour and interrelationships	−0.058	−0.084	−1.047
UP. Unification of persons sharing common interests, attitudes and objectives	Unification of persons sharing common interests, attitudes and objectives	0.202	0.277	4.693***
CC. Confrontation of conflicting interests, attitudes and objectives	Confrontation of conflicting interests, attitudes and objectives	−0.028	−0.041	−0.523
TD. Tolerating “different” persons	Tolerating “different” persons	0.145	0.201	3.448***

R – multiple correlation coefficient; R^2^ – cumulative coefficient of definiteness (coefficient of determination), which shows the part of the dispersion of a dependent variable which is explainable by independent variables; R^2^ revised – based on the sample size and the number of independent variables. Regression is statistically significant when p < 0.001***.

To determine which of the variables have impact on the climate as well as nepotism and favouritism in organisations, two regression equations were created.

OM = 0.512 + 0.180 × MB-N&F + 0.103 × VT - N&F + 0.153 × CI - N&F + 0.285 × EB-N&F + 0.145 × TD-N&F.

The following variables were highlighted as having significant impact on the climate in the organisation: manager’s behaviour, safety and relationships with employees, values and traditions, communication, information sharing, employee behaviour and interrelationships, and tolerating “different” persons. When these variables are improved, while the other remaining ones do not change, the climate in organisations is improved, and vice versa. Still, it must be noted that their impact varies. For instance, the strongest impact can be expected when employee behaviour and interrelationships are improved, also when changes are introduced in management and the managers’ relationships with employees.


N&F=0.705+0.090×C⁢S+0.194×E⁢D+0.202×U⁢P+0.145×T⁢D.

Four variables have been determined to have a significant influence on the diminishment or increase of nepotism in an organisation. They are fears related to the lack of definiteness and safety; organisation being joined by persons who share common interests; unity; and tolerance of “different” persons. When these variables increase (improve) individually, whereas the other remaining variables do not change, the situation related nepotism and favouritism “improves” (i.e., expression of nepotism and favouritism weakens, or at least, does not increase) and vice versa. In terms of strength of impact (from strongest to weakest), in this case, standing out the most are employee groups who share common interests or views and the procedures of employees entering the organisation, working, and exiting the organisation that can be perceived as biassed toward separate individuals.

## Discussion

Good organisational climate, which manifests as individual perception of the working environment, is vitally important for the smooth operation of organisations (Parker et al., 2003; Alwaheeb, 2020; Beus et al., 2020). Nevertheless, evidently, it is impossible to avoid work with relatives and the influence related to this during recruitment (Holm et al., 2017); therefore, these processes must be monitored, and the ways must be sought to reduce the negative impact (Horak, 2018). Neill et al. (2019) have specified that managers who seek employees to be more committed and identify themselves with the organisation have to be sincere and create an atmosphere based on trust, whereas Arasli et al. (2019) revealed that favouritism is related to the violation of the psychological contract and unsafe work climate. Our study shows that nepotism and favouritism are mutually related to climate. Employees react sensitively to biassed behaviour of managers when greater favour is shown toward family members and favourites in the internal processes of the organisation. Importantly, the persons that are labelled as nepots and favourites by the employees stand out from other members of the organisation and are treated in hostile manner as “others.” The view that these persons can receive exceptional favoured treatment from the managers encourages others to monitor them closely. The marked persons are perceived as a group which shares specific connections, carries a threat to personal interests of the non-beneficiaries, and causes the feeling of insecurity. This confirms the results of other research which indicate that nepotism and favouritism are a significant factor which promotes mistrust and insecurity (Daskin, 2013; Arasli et al., 2019). However, our research shows that negative reactions to the group of individuals labelled as nepots and favourites may also be related to subjective prejudices; therefore one cannot reject the impact of rising discriminatory tendencies on the climate due to the intergroup competition related to the organisation’s resources. For instance, Abbink and Harris (2019) have determined that closeness in the group, which can be encouraged simply by labelling, is the main driving force of group favouritism, whereas discrimination outside the group is determined by social distance, conflicts, and competition between different groups. Moreover, Dağli and Akyol (2019), who researched favouritism in education organisations, drew attention to the fact that the existence of groups with different interests stimulates the emergence of discrimination, inequality, or injustice. All of this demonstrates that nepotism and favouritism are a two-way discrimination: first, the privileges granted to groups that share specific connections are perceived as discriminating against the group which is not favoured; second, the group which sees itself as disadvantaged harbours prejudices against the favourites. Also, as shown by the results of correlation and regression analysis, intergroup tension is significantly influenced by the shortcomings of the organisation’s internal communication, which prevent one from achieving the sense of greater definiteness and security. In such a case, both real and imagined threat of nepotism and favouritism can have an impact.

According to Herr et al. (2018), the perception of injustice when making decisions is defined as justice climate, which is determined by individual distress or even somatic disorders. The results of our research show that the procedures of recruitment, career, and dismissal are one of the areas in which significant risk of nepotism and favouritism emerges. When these procedures are evaluated subjectively as more favourable toward persons with connections, they become a source of perception of injustice. For this reason, the impact of nepotism and favouritism on the climate can be described based on the perspective of procedural justice (Colquitt et al., 2001; Tremblay et al., 2010; Chernyak-Hai and Tziner, 2014; Jones and Stout, 2015; Hudson et al., 2019).

### Practical Implications

The results of this study have several consequences on the practice of organisational management because they demonstrate the areas of the organisation’s activities and the mistakes that influence the negative climate. According to Shen et al. (2019), the employees who perceive themselves as less valued and respected as others may reduce their contributions in the organisation. However, even if the employees do not directly associate their achievements and evaluations with the management’s biassed favour toward nepots and favourites, the procedures related to entering the organisation, career, and exiting the organisation require special attention from the managers. The procedures’ transparency and honest application to all employees as well as managers’ ethical behaviour and effective communication policies can serve to decrease intergroup tension and create a positive climate. Such policies can strengthen the employees’ sense of definiteness and safety, and eliminate the reasons for the emergence of hostility against persons who are related to the managers. Due to this, even in the cases when employment of relatives is unavoidable, the negative impact can be reduced.

## Conclusion, Limitation, and Future Research

This research fills the gap in the knowledge on the connections between climate in organisations and nepotism and favouritism, while drawing attention to the mutual interaction between these phenomena. The authors of the research sought to present the empirical contribution while describing which components of the organisation’s climate have an influence on the expression of nepotism and favouritism. The obtained results support the view that nepotism and favouritism have a negative effect on the organisation’s climate, but this effect is not unambiguous. Moreover, several important factors were singled out that are related to favour toward relatives and that cause intergroup tension, whose regulation could reduce the negative impact of nepotism and favouritism on the climate. Even though nepotism and favouritism are considered as a natural phenomenon, the view is maintained that its impact on climate in the organisation can be managed.

### Limitations

Most of the respondents in this study were persons who are aged up to 40 and have higher university and non-university education. Education may have had an impact on the questionnaire’s better understanding, though the results do not fully reflect those areas of professional activity that have lower education requirements. The fact that the research was conducted in a single country limits the possibility of wider conclusions. In addition, as homogeneous groups have not formed, no calculations were performed in this study not only by education, but also by age, seniority, and the origin of corporate capital. However, it cannot be ruled out that these variables could have had certain influence, as the enterprise’s policy with regard to nepotism and favouritism depend on both the attitude of the owners of the enterprise itself and the traditions established in that country (e.g., Safina, 2015; Vveinhardt and Sroka, 2020). Nepotism and favouritism may depend on variables such as gender, age, or education of employees (Sandström and Hällsten, 2008; Akuffo and Kivipõld, 2019; Gorji et al., 2020). For this reason, for example, the responses of women (74%) could have influenced the discrimination variable due to managers’ poorer attitude toward them (Sandström and Hällsten, 2008); therefore, additional research would be useful in the future. Despite the said limitations, the main focus of our work was to demonstrate how the existence of nepotism and favouritism affected or did not affect individual variables of the organisational climate in general.

### Future Research

Aside from the fact that nepotism and favouritism indeed usually manifest in recruitment processes (Padgett et al., 2019), one cannot also reject the additional influence of prejudices, which is related to the views prevalent in society. For this reason, it could be meaningful to conduct a more detailed examination of the extent to which prejudices have influence on views toward nepotism and favouritism and how they affect the climate. Interestingly, the respondents reacted very sensitively to the processes of entering the organisation, leaving it, and rising the career ladder, even though the employee assessment procedures were not significantly related to climate. Due to this, these issues could be researched in more detail. Future research should also assess the role of the origin of the enterprise’s capital; i.e., shareholders’ role, in shaping the policy of these enterprises with regard to nepotism and favouritism. It is appropriate to perform the analysis of demographic variables, determining how the attitude of different generations of employees to the patronage of relatives and favourites differs or coincides.

### Directions for Future Research

For future research, the questionnaire is planned to be shortened and translated to the languages of the neighbouring countries, while surveys are planned to be conducted in target groups, and re-testing is intended to be performed later.

## Data Availability Statement

The raw data supporting the conclusions of this article will be made available by the authors, without undue reservation.

## Ethics Statement

Ethical review and approval was not required for the study on human participants in accordance with the local legislation and institutional requirements. Written informed consent for participation was not required for this study in accordance with the national legislation and the institutional requirements.

## Author Contributions

JV contributed to conception and design of the study and performed the statistical analysis. JV and RB wrote the first draft of the manuscript and wrote sections of the manuscript. All authors contributed to manuscript revision, read, and approved the submitted version.

## Conflict of Interest

The authors declare that the research was conducted in the absence of any commercial or financial relationships that could be construed as a potential conflict of interest.

## Publisher’s Note

All claims expressed in this article are solely those of the authors and do not necessarily represent those of their affiliated organizations, or those of the publisher, the editors and the reviewers. Any product that may be evaluated in this article, or claim that may be made by its manufacturer, is not guaranteed or endorsed by the publisher.
